# Exercise-induced oxidative and hematological responses in adolescent female swimmers during the luteal phase

**DOI:** 10.3389/fphys.2026.1705876

**Published:** 2026-04-22

**Authors:** Justyna Cichoń-Woźniak, Hanna Dziewiecka, Joanna Ostapiuk-Karolczuk, Marta Mydłowska, Małgorzata Szymańczuk, Anna Skarpańska-Stejnborn

**Affiliations:** Poznan University of Physical Education, Faculty of Physical Culture in Gorzów Wielkopolski, Department of Biological Sciences, Gorzów Wielkopolski, Poland

**Keywords:** adolescents, athletes, blood smear, menstrual cycle, redox balance

## Abstract

**Introduction:**

Physiological response to exercise in young female athletes is understudied, especially regarding how the luteal phase of the menstrual cycle influences performance in demanding sports such as swimming. Therefore, this study aimed to evaluate the post-exercise cortisol response, oxidative stress markers, and hematological parameters following a moderate-intensity exercise test in trained adolescent female swimmers.

**Materials and methods:**

Eighteen swimmers (aged 12–16 years) performed a swimming test (800m + 200m + 50m) during their rigorously verified luteal phase. Blood samples were collected before exercise, immediately after exercise, and after 3 hours of recovery. It was registered retrospectively on clinicaltrials.gov under NCT06903195 (March 17, 2025).

**Results:**

Moderate-intensity exercise significantly increased 8-isoprostane levels (100.70 (40.50-131.00) vs 145.10 (99.16-190.40), p<0.05), while cortisol and 4-hydroxynonenal levels decreased after 3 hours. Microscopic blood smear analysis showed erythrocyte anisocytosis and poikilocytosis, toxic granulation in granulocytes, and activated lymphocytes.

**Conclusion:**

The evaluation of young female swimmers during the luteal phase of their menstrual cycle revealed stable and subsequently decreasing cortisol levels, indicating that the exercise did not elicit a significant endocrine stress response. In contrast, the same protocol resulted in elevated levels of 8-isoprostanes. This latter finding demonstrates that the moderate-intensity exercise was sufficient to induce exercise-induced oxidative stress in this specific group of athletes.

## 1 Introduction

Women are more susceptible to stress-related disorders than men, particularly during adolescence. This suggests that interactions between female sex hormones and cortisol are key factors in the stress response, yet research involving female participants (especially adolescents) remains limited and often contradictory (Sung et al., 2014; Hamidovic et al., 2020; Oleka, 2020). Complexity of the menstrual cycle is often cited as a key barrier to inclusion, leading to significant gaps in sports science regarding how cycle-specific hormonal shifts influence post-workout biochemical and morphological recovery (Hamidovic et al., 2020). Investigating these physiological changes is essential for developing personalized training programs aimed at optimizing athletic performance and minimizing the risk of injury in young female athletes.

The menstrual cycle significantly modulates physical performance and recovery. While the follicular phase often facilitates more effective strength training and improved adaptability (Sung et al., 2014; Oleka, 2020), the luteal phase presents distinct physiological challenges. Specifically, increased levels of allopregnanolone, which is a progesterone metabolite, have been associated with altered cortisol dynamics during this phase (Hamidovic et al., 2020). Furthermore, the luteal phase is characterized by a shift toward catabolic metabolism and heightened reactive oxygen species (ROS) production (Sung et al., 2014; Cagnacci et al., 2023). While the follicular phase is associated with higher estrogen levels that may increase the expression of antioxidant enzymes (Yu et al., 2012), the luteal environment is often accompanied by increased lipid peroxidation and a concomitant decrease in superoxide dismutase (SOD) activity (Lu et al., 2018). This suggests a heightened baseline oxidative challenge. Consequently, exercise-induced stress during the luteal phase may lead to reduced recovery and impaired adaptation to training stimuli (Chica-Latorre et al., 2025), indicating that the body’s response to post-workout oxidative stress varies significantly across different phases of the menstrual cycle.

Coexisting oxidative stress in the body can lead to lipid peroxidation (LPO) in cell membranes, processes that are exacerbated by hormonal fluctuations. LPO produces numerous secondary products, most notably 4-hydroxynonenal (4-HNE) and 8-isoprostanes (8-iso). 4-HNE is a highly cytotoxic aldehyde that can inactivate antioxidant enzymes and trigger cell apoptosis (Li et al., 2022), while 8-iso serves as a sensitive gold-standard marker of endogenous oxidative damage (Milne, 2017a; Meera et al., 2020a). Evidence suggests that cortisol plays a critical role in this process by stimulating ROS production and depleting glutathione (GSH) levels (de Oliveira et al., 2022; Signorello et al., 2024; Zhang and Kong, 2025). While cortisol-induced LPO has been documented in platelets (Karolczak et al., 2023), its synergistic effect with the luteal phase on 4-HNE and 8-iso levels, and the resulting structural integrity of other blood cells, remains inadequately studied in adolescent athletes. Oxidative damage can also alter the structure and mechanical properties of the erythrocyte membrane, including its deformability, which raises the risk of hemolysis (Orrico et al., 2023). As a result, the presence of morphologically altered erythrocytes in the blood smear may occur, serving as an important indicator not only of oxidative stress but also of potential functional issues in the cardiovascular system (K.T. et al., 2022). Consequently, qualitative assessment of peripheral blood smears under a light microscope provides a valuable diagnostic tool for detecting subtle deviations in erythrocyte and leukocyte morphology (Krüger and Mooren, 2014; Karolczak et al., 2023). This approach allows for the evaluation of cellular responses to the synergistic effects of exercise and hormonal fluctuations. This study addresses the research gap by focusing on adolescent female swimmers, who may show increased sensitivity to stress stimuli during maturation (Hackney, 2006; Athanasiou et al., 2023).

This study aimed to examine post-workout cortisol levels and specific oxidative stress markers in young female swimmers during the luteal phase of their menstrual cycle after a moderate-intensity workout. It was hypothesized that a standardized swimming test would lead to a significant elevation in oxidative stress markers, specifically increased lipid peroxidation and pronounced qualitative alterations in erythrocytes, in young female swimmers during the luteal phase. Furthermore, we specifically anticipated that these redox imbalances would positively correlate with post-exercise cortisol concentrations, suggesting a link between the intensity of the hormonal stress response and the degree of oxidative damage.

## 2 Materials and methods

### 2.1 Participants

This observational study was conducted between October and December 2023 at the “Słowianka” Sport and Rehabilitation Center in Gorzów Wielkopolski. A total of 18 female trained swimmers attending the Sports Championship School Complex, aged 12–16 years, participated in the study. Before exercise, the anthropometric parameters were measured using an electronic scale to the nearest 0.05 kg (Tanita BC-980 MA, Tanita Corporation, Tokyo, Japan), while body height was measured using a stadiometer Seca 213 (Seca, Hamburg, Germany). The results are shown in **Table 1**. The participants were instructed to consume a light meal consisting of protein, carbohydrates, and minimal fat (e.g., cereal with milk) on the day of the study. The meal had to be eaten at least 2 hours before the examination began.

**Table 1:** Basic characteristics of study participants (N = 18).

Parameters	Median (IQR)
Age (years)	12–16 (min-max range)
Body mass (kg)	55.25 (52.25-60.18)
Height (cm)	163.00 (160.80-171.30)
Training internship (years)	6.00 (4.75-6.00)

### 2.2 Inclusion and exclusion criteria

Inclusion criteria were: a valid medical certificate of fitness for competitive sports (issued by a sports medicine physician), consent from the legal guardian of the underage athlete to participate in the study, at least three years of training experience, and completion of the scheduled swimming test in the study. The exclusion criteria were: antibiotic therapy, supplementation, use of any drugs or oral hormonal contraceptives, and health problems within the last month.

### 2.3 Study design

**Figure 1:**
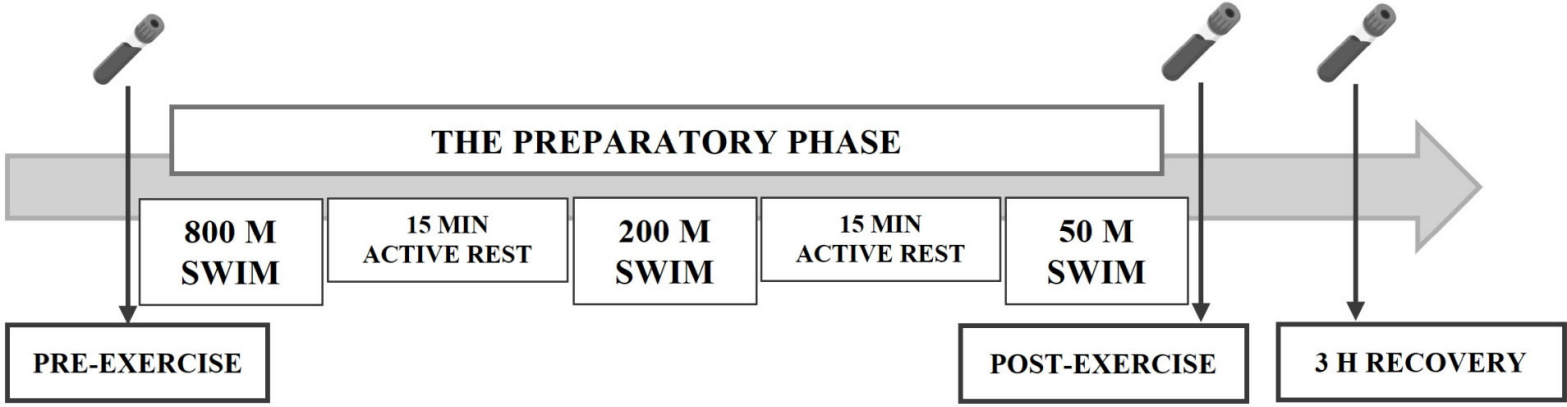
Study design.

In this study, the athletes underwent swimming tests in front crawl (800m + 200m + 50m) with 15 minutes of active rest in the water (**Figure 1**). Basic characteristics of the exercise are shown in **Table 2**. Exercise intensity was characterized using objective post-exercise indicators, including heart rate and blood lactate concentration. Based on these parameters, the protocol was classified as moderate-intensity exercise. The swim test was conducted during the preparatory phase. Test date was individually selected for each female athlete, based on the training period and the monitored menstrual cycle (luteal phase). The selection of this test protocol was based on previous research (Emami, 2019) demonstrating its effectiveness in eliciting significant physiological responses, including marked changes in oxidative stress and cortisol markers, in elite male swimmers. Based on previous findings, the protocol was considered sufficiently demanding to elicit measurable physiological responses in the studied cohort of adolescent female swimmers.

**Table 2:** Basic characteristics of exercise (N = 18).

Parameters	Median (IQR)
800 m swim	
HR_max_ (bpm) HR_mean_ (bpm)	193.00 (189.50-198.50) 173.00 (170.00-182.00)
Test duration (s)	701.60 (671.0-767.70)
200 m swim	
HR_max_ (bpm) HR_mean_ (bpm)	192.00 (183.00-197.00) 178.00 (174.00-184.50)
Test duration (s)	158.00 (153.40-172.80)
50 m swim	
HR_max_ (bpm) HR_mean_ (bpm)	177.00 (175.50-188.50) 161.00 (156.50-171.00)
Test duration (s)	33.79 (32.60-36.61)
LA levels:	
Pre-exercise	0.97 (0.91-1.12)
Post-exercise	4.77 (3.69-6.22)

HRmax (maximal heart rate), LA (lactic acid).

Menstrual cycle was tracked through three stages of control: a six-month cycle history before the first stress test, ovulation testing, and hormonal level assessments, including progesterone, 17-β estradiol, luteinizing hormone, and follicle-stimulating hormone. The swimmers monitored their menstrual cycle daily using the Period Tracker Period Calendar mobile application (Abishkking Limited, Hong Kong, China). In the second stage, ultra-sensitive ovulation tests (Diather, Gdańsk, Poland) were used to track the cycle. In the third stage, on the day of the exercise test, sex hormone concentrations were determined via ELISA (enzyme-linked immunosorbent assay) using diagnostic kits (DRG Instruments GmbH, Germany). Detailed description of the control methodology is the subject of a separate publication currently in preparation. However, the raw data supporting this control, including all hormone measurements, are openly available in the data repository at: (https://doi.org/10.18150/EQQESR).

The study was conducted in accordance with the principles of the Declaration of Helsinki and approved by the Bioethical Committee at Poznan University of Medical Sciences, Poland (decision no. 538/22 in 2022, with amendments 221/23 in 2023). It was registered retrospectively on clinicaltrials.gov under NCT06903195 (March 17, 2025). All procedures and potential risks were discussed with the participants before the study. Informed consent was obtained from all participants before they participated in the study and their parents or legal guardians.

Daily records were maintained to document the characteristics of the training profile, including intensity, volume (measured in minutes), and type, as detailed in **Table 3**.

**Table 3:** Daily training program before the swimming test.

Training	I	Days II	Before III	Test IV	V
Time swum, min/day	180	150	110	220	115
Distance swum, m/day	6300	5900	3200	7300	3500
Moderate endurance swimming training time, min/day	180	150	95	190	70
Extensive endurance swimming training time, min/day	0	0	15	20	30
Training for force development, min/day	0	30	0	0	50
Very high intensity endurance rowing training time, min/day	0	0	0	10	15
Unspecific training (running, etc.), min/day	40	40	25	50	60
Total training time, min/day	220	220	135	270	225

### 2.4 Material collection and blood analysis

Blood samples were taken from the cubital vein at three time points: before test (pre-exercise), one minute after the end of the test (post-exercise), and after a 3-hour recovery period (3h recovery). During blood collection, it was considered that cortisol concentrations show diurnal variation and peak in the morning hours (6:00 a.m. - 9:00 a.m.). Accordingly, the first blood draw took place around 6:00 a.m., followed by a post-exercise sample around 7:00 a.m., while the last collection was around 10:00 a.m. The collection times were kept consistent for all female athletes.

Polyethylene tubes containing dipotassium ethylenediaminetetraacetic acid (EDTA-K2) anticoagulant were used to perform the blood smear. Small drop of blood was placed on a slide, smeared, air-dried, stained using the May Grunwald-Giemsa method. The smears were then evaluated under a microscope (Opta Tech, Warsaw, Poland) by two qualified laboratory diagnosticians. Blood for cortisol, 4-HNE, and 8-iso measurement was collected in polyethylene clotting activator tubes. Upon sampling, samples were left at least 45 minutes to clot, and then were centrifuged at 3000 rpm for 10 min to separate serum. Serum aliquots were stored in tubes (Eppendorf SE, Hamburg, Germany), at -80 °C.

Biochemical parameters (cortisol, 4-HNE, and 8-iso) were measured from the extracted serum using ELISA (DRG Instruments GmbH, Germany, for cortisol; SunRed Biotechnology Company, Shanghai, China, for 4-HNE and 8-iso) according to the test manufacturer’s instructions. The assay ranges were 3.6–2208 nmol/L for cortisol, 3–600 pg/ml for 4-HNE, and 5–1000 ng/L for 8-iso. A quality control assessment of the assays was performed by evaluating their repeatability and reproducibility. The obtained coefficient of variation (CV%) values were <10 for intra-assay and <15 for inter-assay measurements. The lactate (La) concentration was determined immediately after collection from capillary blood using dedicated reagents and the Vario Photometer II portable biochemical analyzer (Diaglobal, Berlin, Germany). Internal Quality Control (QC) was performed to ensure the reliability of La measurements, utilizing three levels of commercial control material provided by the manufacturer (target values: 2 nmol/L, 4 nmol/L, 10 nmol/L). All obtained mean results for the QC materials fell within the specified confidence intervals. The measured CV% for all assays was low (<3.5%).

### 2.5 Sample size

Representative study population - sample size was calculated using G-power software (Faul et al., 2007). Specifically, we used an effect size derived from the study by Ostapiuk-Karolczuk et al. (2025) in which cortisol changes were analyzed (Ostapiuk-Karolczuk et al., 2025). This effect size was selected because the cited study examined exercise-induced cortisol responses under comparable experimental conditions. Accordingly, we conducted *a priori* power analysis in G*Power for repeated-measures ANOVA (within factors), using the following parameters: effect size (f) = 0.53 and Power (1 – β) = 0.8. This yielded a required minimum sample size of 12 participants, which we exceeded by recruiting 18.

### 2.6 Statistical analyses

All statistical analyses and data visualizations were carried out using GraphPad Prism version 10 (GraphPad Software, Boston, USA). Descriptive statistics, expressed as median and interquartile range (IQR), were used to summarize the data and illustrate patterns across the three measurement time points: before exercise, immediately after exercise, and following a 3-hour recovery.

Outlier detection was performed using the ROUT method (Q = 1%), and no statistical outliers were identified. Given the limited number of participants and deviations from normality assumptions, the Friedman test was applied to evaluate differences across repeated measurements, followed by Dunn’s *post hoc* test for pairwise comparisons. For binary (0/1) variables assessed at multiple time points, the Wilcoxon matched-pairs signed-rank test was used for pairwise comparisons. Cohen’s d was calculated as a measure of effect size, with interpretation based on Cohen’s criteria: small (0.2), moderate (0.5), and large (0.8). Pearson’s coefficient of linear correlation was computed for correlation analysis (Cohen, 1988).

## 3 Results

### 3.1 Hormonal analysis

**Figure 2:**
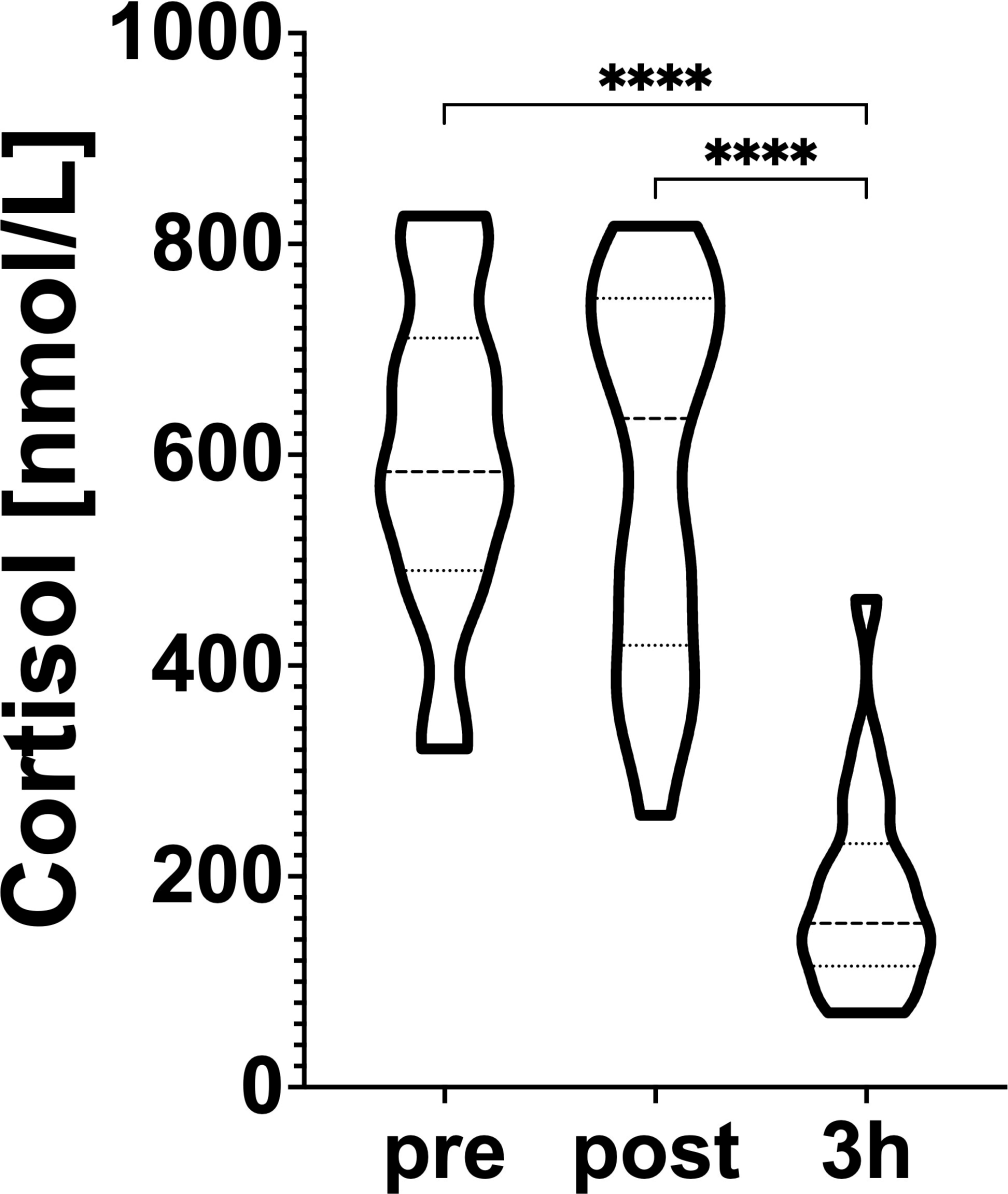
The effect of exercise on cortisol serum concentration. PRE (pre-exercise), POST (post-exercise), REC (3h recovery). Significant differences: ****p < 0.0001.

Cortisol serum concentration slightly increased after exercise and then significantly decreased in the 3h recovery (*p<*0.0001, Cohen’s d = 3.35, pre-exercise vs 3h recovery; *p<*0.0001, Cohen’s d = 2.89, post-exercise vs 3h recovery) (**Figure 2**).

### 3.2 Markers of lipid peroxidation

**Figure 3:**
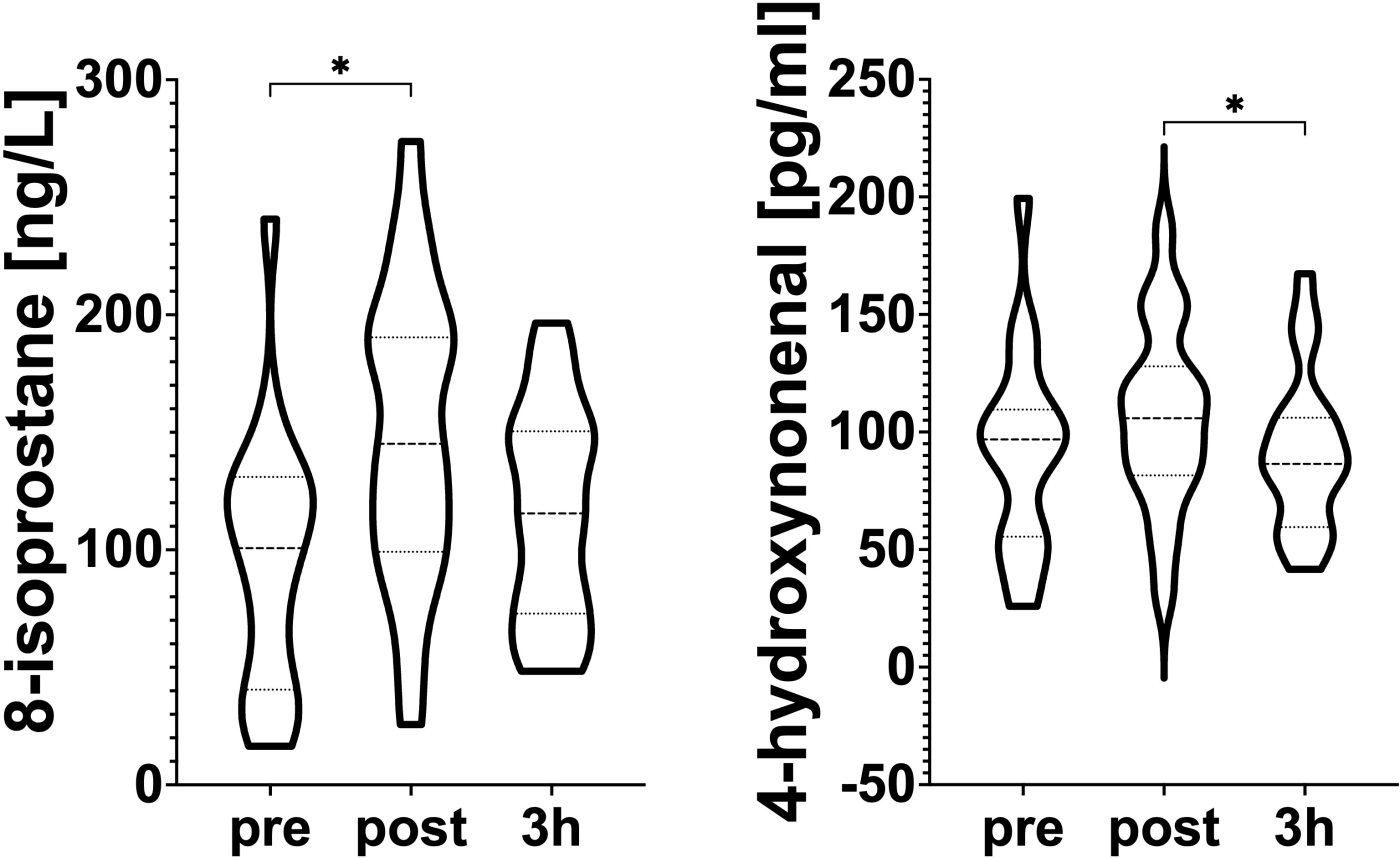
The effect of exercise on 4-HNE, and 8-iso serum concentration. PRE (pre-exercise), POST (post-exercise), REC (3h recovery). Significant differences: *<0.05.

Serum 8-iso, and 4-HNE levels are presented in **Figure 3**. 8-iso significantly increased immediately after exercise (*p*< 0.05, Cohen’s d = 0.88, pre-exercise vs. post-exercise). 4-HNE serum concentration significantly decreased in the 3h recovery (p<0.05, Cohen’s d = 0.00, pre-exercise vs 3h recovery).

### 3.3 Microscopic evaluation of blood smear

Frequency of specific morphological changes was calculated as a percentage within the study group based on microscopic evaluation of peripheral blood smears (**Table 4**). Anisocytosis was present in 39% of participants both at rest and immediately after exercise. After 3 hours of recovery, its occurrence decreased to 33%. Poikilocytosis was observed in only 6% of participants at rest, increasing to 28% immediately after exercise and to 33% after 3 hours of recovery. Toxic granulation in the cytoplasm of neutrophils was seen in 6% of individuals at rest. This percentage increased to 28% after exercise and then significantly rose to 39% following recovery. No statistically significant differences were found in the incidence of the changes evaluated.

**Table 4:** Percentage presence of changes in microscopic blood smear analysis.

Feature	Pre	Post	Rec
Anisocytosis [%]	39	39	33
Poikilocytosis [%]	6	28	33
Toxic granulation [%]	6	28	39^b^

PRE (pre-exercise), POST (post-exercise), REC (3h recovery). ^b^ Pre-exercise vs. recovery (*p* < 0.05).

The effect of e exercise on leukocytes is presented in **Table 5**. Neutrophil count was significantly increased after 3h of restitution (*p<*0.0001, Cohen’s d = 1.56, pre-exercise vs. 3h recovery; *p=*0.0002, Cohen’s d = 1.61, post-exercise vs. 3h recovery). Number of lymphocytes significantly decreased after 3 h of restitution (*p<*0.0001, Cohen’s d = 1.54, pre-exercise vs. 3h recovery; *p<*0.0001, Cohen’s d = 1.38, post-exercise vs. 3h recovery), and reactive lymphocytes increased after exercise and recovery (*p=*0.0138, Cohen’s d = 1.00, pre-exercise vs. after-exercise; *p=*0.0007, Cohen’s d = 1.14, pre-exercise vs. 3h recovery). Monocyte count was significantly decreased (*p=*0.0061, Cohen’s d = 0.96, pre-exercise vs. 3 h recovery). Eosinophil count was significantly decreased after 3h of restitution (*p=*0.0005, Cohen’s d = 1.59, pre-exercise vs. 3h recovery; *p=*0.0179, Cohen’s d = 1.24, post-exercise vs. 3h recovery). No significant changes were observed in basophil count.

**Table 5:** The effect of intense exercise on leukocytes.

Leukocyte parameters	Pre	Post	Rec	Cohen’s d
NEU [%]	49.00 [43.50-56.75]	50.00 [45.25-54.25]	62.50 (59.50-66.00)^b, c^	1.56^b^; 1.61^c^
LYM [%]	43.50 [38.75-46.25]	41.00 [34.50-50.00]	31.00 [27.75-33.25]^b, c^	1.54^b^; 1.38^c^
REACTIVE LYM [%]	0.50 (0.00-2.25)	3.00 (1.75-4.25)^a^	2.50 (2.00-5.00)^b^	1.00^a^; 1.14^b^
MON [%]	3.00 [2.00-4.25]	2.00 (1.00-3.00)	1.00 (1.00-2.25)^b^	0.96^b^
EOS [%]	3.00 [2.00-4.00]	3.00 [1.00-4.25]	1.00 [0.00-2.00]^b, c^	1.59^b^; 1.24^c^
BASO [%]	0.00 [0.00-0.00]	0.00 [0.00-0.00]	0.00 [0.00-0.00]	

PRE (pre-exercise), POST (post-exercise), REC (3h recovery). NEU (neutrophils), LYM (lymphocytes), MON (monocytes), EOS (eosinophils), BASO (basophils). ^a^Pre-exercise vs. post-exercise, ^b^Pre-exercise vs. recovery, ^c^Post-exercise vs. recovery (p < 0.05).

## 4 Discussion

The present study provides new insights into the physiological responses of young female swimmers to a standardized bout of moderate-intensity exercise performed during the luteal phase of the menstrual cycle. Despite the anticipated activation of the hypothalamic–pituitary–adrenal axis, post-exercise cortisol concentrations remained stable, suggesting that the applied exercise load did not trigger a substantial endocrine stress reaction in this cohort. In contrast, a significant increase in 8-isoprostanes was observed, indicating that the same exercise stimulus was sufficient to elicit oxidative stress. This divergence between hormonal and oxidative responses highlights the complexity of exercise-induced physiological adaptations and underscores the importance of considering menstrual cycle phase when interpreting stress- and redox-related biomarkers in female athletes.

The study found no statistically significant changes in serum cortisol levels immediately after exercise in analyzed swimmers (**Figure 2**). Results of previous studies on the cortisol response to exercise remain inconclusive. Literature reports indicate both a post-exercise increase in the concentration of this hormone (Paccotti et al., 2005; Faul et al., 2007; Hough et al., 2011; Popovic et al., 2019), a decrease (Hough et al., 2013; Sullivan et al., 2019; Taha and Mounir, 2019), and no significant change (Koc, 2018). These discrepancies have prompted many authors to investigate the reasons for the varying responses. It has been suggested that the increase in cortisol secretion mainly occurs in response to high-intensity exercise. In contrast, low- and moderate-intensity exercise does not cause significant changes in its levels (VanBruggen et al., 2011; Anderson et al., 2019).

However, Ntovas et al. (2022) compared post-workout cortisol levels in female athletes training in handball and swimming, showing an increase in this hormone among handball players, with no significant change in swimmers (Ntovas et al., 2022). Talebi et al. (2013) also noted a notable decrease in cortisol levels after exercise among swimmers. This indicates that, in addition to the intensity of the physical load, the specific nature of the sport practiced may also influence the cortisol response (Talebi et al., 2013). The lack of significant post-exercise changes in cortisol is likely linked to the moderate exercise intensity, which is supported by only a moderate rise in lactate levels. However, the specific nature of swimming, which involves distinct hemodynamic conditions, might also alter the hormonal response (Ntovas et al., 2022).

Almási et al. (2021) examined young athletes participating in water sports such as swimming and water polo. Boys’ cortisol levels rose after exercise. In contrast, the girls’ group had higher resting cortisol levels and showed no significant change in response to exercise. Authors mention that the studied female athletes were in different phases of the menstrual cycle, which could have influenced the results (Almási et al., 2021).

A significant decrease in cortisol levels was observed after a 3-hour recovery period (**Figure 2**). This decline, however, is primarily interpreted as an effect of the natural diurnal rhythm (Segerstrom et al., 2017). While resting and immediate post-exercise measurements were taken within the morning peak secretion window, the 3-hour recovery measurement fell outside this time frame. Therefore, the observed decrease is mainly due to the natural decline in cortisol levels throughout the day. The study’s primary goal was to assess the pattern of post-exercise changes, and the role of this hormone in recovery requires further investigation.

In this study, 4-HNE concentration decreased after three hours, while 8-iso concentration increased significantly post-exercise (**Figure 3**), confirming lipid peroxidation in trained swimmers. Higher sensitivity and specificity of 8-iso compared to other redox status parameters may explain the divergence in these results (l’yasova et al., 2012; Labuschagne et al., 2013; Milne, 2017a; Milne, 2017b; Meera et al., 2020b). While some reports suggest that swimming may boost antioxidant defenses (Inal et al., 2001; Ookawara et al., 2003; Morissette et al., 2016; Sánchez Chapul et al., 2022; Wu et al., 2025), our results indicate the presence of oxidative damage during the luteal phase, likely linked to hormonal changes.

Santos-Silva et al. (2001) demonstrated that adolescents who train in swimming experience higher levels of oxidative stress compared to inactive peers (Santos-Silva et al., 2001). Similar results were reported by Gougoura et al. (2007), who observed an increase in lipid peroxidation in trained children who participated in swimming compared to the non-training group (Gougoura et al., 2007). In contrast, Tauler et al. (2008) observed a gender-dependent effect of swimming sessions on oxidative damage levels in adolescents, showing that boys were more susceptible to these changes than girls (Tauler et al., 2008).

In growing youth, the flow of oxygen to working muscles might differ from that of adults, potentially influencing the response to exercise-induced oxidative stress (Santos-Silva et al., 2001; Cooper et al., 2004). Although some literature suggests that the antioxidant defense system in youth undergoes physiological adaptation (Gougoura et al., 2007), our data do not allow for a definitive characterization of the antioxidant system status in this cohort. Furthermore, results of the present study, in which no post-exercise increase in cortisol was observed alongside a rise in 8-iso, highlight the complexity of the interaction between hormones and oxidative stress parameters. A review of the literature shows that hormones such as progesterone, estradiol, melatonin, and insulin have antioxidant properties, while catecholamines, corticosteroids, and thyroid hormones increase free radical production and lead to oxidative stress (Chainy and Sahoo, 2020). Therefore, caution should be taken when interpreting the relationship between a single hormone and indicators of oxidative status, especially considering the dynamic hormonal changes occurring in women.

Increased occurrence of poikilocytosis in the qualitative assessment of blood smears after the swim test might suggest exercise-related damage to cell membrane integrity (**Figure 3**; **Table 4**). Shape abnormalities observed in erythrocytes may result from heightened lipid peroxidation caused by oxidative stress (Obeagu et al., 2024). Changes in the composition and structure of erythrocyte cell membrane phospholipids are likely to decrease erythrocyte elasticity and stability (Barshtein et al., 2023). The few changes in blood cell morphology were not statistically significant; therefore, this study cannot definitively confirm whether oxidative stress contributes to poikilocytosis. This may be due to the relatively low lactate levels, which suggest moderate exercise intensity, likely causing only minor oxidative damage.

Alternatively, the high cumulative training volume during the preparatory phase might have influenced baseline erythrocyte stability, rather than the single exercise test itself. This preliminary interpretation is consistent with the biomarker profile, which showed an increase in the sensitive marker 8-iso, while the level of 4-HNE decreased after 3 hours of recovery. A decrease in lymphocyte and monocyte counts observed in this study after three hours of recovery aligns with literature reports indicating that lymphocyte counts drop below resting levels as early as 30 minutes after exercise ends and typically take 4–6 hours to return to baseline (Peake et al., 2017; **Table 5**). This phenomenon is thought to result from the movement of lymphocytes and monocytes from the blood to other tissues, possibly due to glucocorticoids (Peake et al., 2017). Nevertheless, based on our results, we cannot determine the effect of cortisol on this process because its concentration fluctuates diurnally during the regenerative recovery period (Peake et al., 2017).

**Figure 4:**
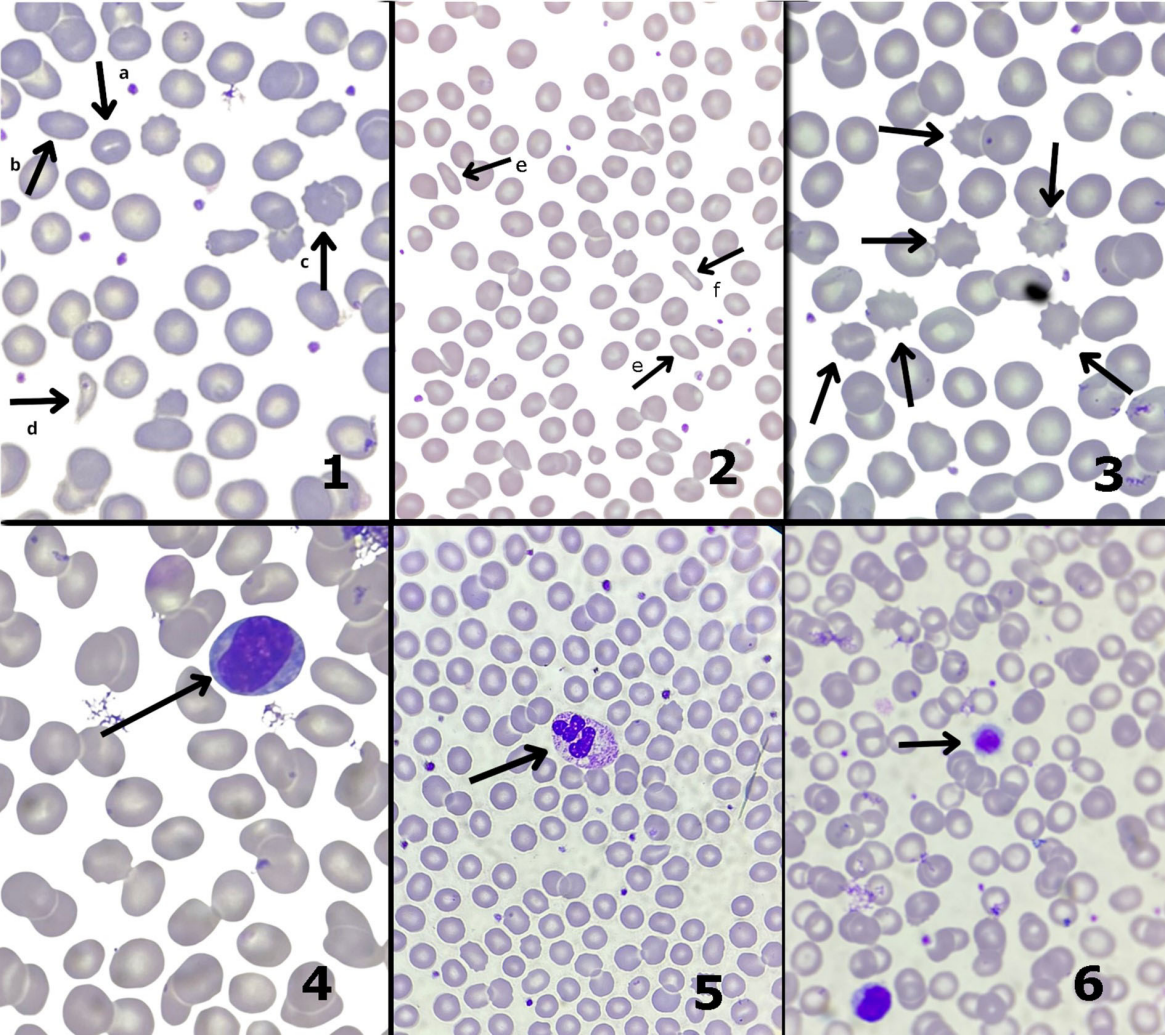
Blood cell morphology. 1 poikilocytosis RBC; (a, stomatocyte; b, elliptocyte; c, echinocyte; d, schistocyte), 2 poikilocytosis RBC (e, elliptocyte; f, pencil cell), 3 poikilocytosis RBC (echinocytes), 4 reactive lymphocytes, 5 toxic granulation in a neutrophil, 6 giant platelet (100x, oil).

The increase in reactive lymphocytes and toxic granulation observed in this study (**Figure 4**; **Tables 4**, **5**) might be tentatively linked to inflammatory mechanisms and the perturbation of the myelopoiesis system (Ahnach et al., 2020). Given the qualitative nature of these findings and the limited sample size, these results indicate a need for further research using higher-intensity protocols to better understand the mechanisms leading to morphological changes in RBCs.

Research on the effect of the menstrual cycle phase on exercise performance and post-exercise biochemical changes in athletes remains limited. These studies are also characterized by high variability. This is due to both methodological factors and small sample sizes (Meignié et al., 2021). Existing evidence suggests that the luteal phase may exhibit reduced adaptation to oxidative stress in response to training stimuli (Meignié et al., 2021). Our study also confirms this finding. Nevertheless, some other works indicate that hormonal changes do not affect anaerobic performance, starting speed, or anaerobic endurance (Wiecek et al., 2016; Carmichael et al., 2021). Another body of research suggests decreased exercise efficiency in the follicular phase compared to the luteal phase (McNulty et al., 2020). Therefore, there is no clear determination of the cycle phase’s impact on sports results. This highlights the urgent need for additional research: specifically, long-term and prospective studies. Consequently, training individualization is crucial for elite athletes to optimize sports outcomes (McNulty et al., 2020). This individualization should be maintained until more consistent evidence allows for the creation of precise recommendations regarding the adjustment of training loads to the menstrual cycle phases (Meignié et al., 2021).

## 5 Conclusion

Moderate-intensity exercise in young female swimmers during the luteal phase did not alter cortisol concentrations, indicating a limited endocrine stress response. However, the same exercise bout significantly increased 8-isoprostanes, confirming that the stimulus was sufficient to induce oxidative stress. This divergence between hormonal and oxidative reactions highlights the complexity of physiological regulation in female athletes and the importance of accounting for menstrual cycle phase when interpreting post-exercise biomarkers.

Several limitations of the present study should be acknowledged. First, the study’s focus on participants exclusively in the luteal phase limits the generalizability of the results to the entire menstrual cycle. Future research should include athletes across different menstrual phases and sports, while also considering age- and maturation-related differences in antioxidant capacity. Addressing these gaps, along with employing higher-intensity exercise protocols, is necessary to clarify the role of hormonal regulation in redox responses among adolescent female athletes. A limitation of the present study is the relatively small sample size, and therefore the findings should be interpreted with caution.

## Data Availability

The datasets presented in this study can be found in online repositories. The names of the repository/repositories and accession number(s) can be found in the article/supplementary material.
